# Supplementary material for: Effects of Parkinson’s disease and dopamine on digit span measures of working memory
Source: Psychopharmacology (Berl). 2018 Oct 12;235(12):3443–50. doi: 10.1007/s00213-018-5058-6 (PMC6267128; doi:10.1007/s00213-018-5058-6)
Supplement: Supplementary file 1 — 1 The exclusion criteria for Experiment 2, based on the drugs given to participants. (DOCX 17 kb) [file 213_2018_5058_MOESM1_ESM.docx]

Supplementary Materials 1:

Effects of Parkinson’s disease and dopamine on digit span measures of working memory

*Psychopharmacology*

John Patrick Grogan^1^, Lisa Emily Knight^2^, Laura Smith^1^, Nerea Irigoras Izagirre^1^, Alexandra Howat^1^, Brogan Elizabeth Knight^3^, Anastasia Bickerton^1^, Hanna Kristiina Isotalus^1^, Elizabeth Jane Coulthard^1,3^.

1. University of Bristol
2. University Hospitals Bristol
3. North Bristol NHS Trust

Correspondence to: John Grogan. Bristol Brain Centre, Elgar House, Southmead Hospital, Bristol, UK, BS10 5NB. +44 (0)1174148186. [John.grogan@bristol.ac.uk](mailto:John.grogan@bristol.ac.uk).

# Exclusion Criteria for Experiment 2

Experiment 2 recruited 35 healthy older adults for a placebo-controlled drug study. On top of the standard inclusion criteria listed in the manuscript (65+ years old, normal/corrected hearing and vision, no neurological disorders, no dopaminergic medication) there were also several exclusion criteria based on the safety information for the drugs and placebo. These are listed here.

Participants must not have:

- any neurological problems (e.g. stroke, dementia, cerebrovascular disease)
- any psychiatric problems, including psychosis, depression, schizophrenia, anxiety disorder
- a known sensitivity to levodopa, benserazide, domperidone or vitamin C
- lactose intolerance, galactosemia or glucose/galactose malabsorption
- Huntington's Chorea
- Intention tremor
- Prolactin-releasing pituitary tumour
- glaucoma
- a history of malignant melanoma
- a suspicious, undiagnosed skin lesion
- peptic ulcers
- diabetes
- osteomalacia
- severe endocrine, hepatic, renal, pulmonary or cardiac problems
- electrolyte disturbances
- prolongation of cardiac conduction intervals

Participants must not be taking:

- any medications that affect the brain
- any dopaminergic medications
- any serotonergic medications
- any cholinergic medications (anti-cholinergic or cholinesterase inhibitors)
- any noradrenalinergic/norepinephrinergic medications
- Monoamine oxidase inhibitors (MAO inhibitors)
- any blood pressure medications
- ferrous sulphate
- any opoids
- any sympathomimetics (e.g. amphetamines, adrenaline/epinephrine)
- diazepam
- any neuroleptics/antipsychotic medications
- any CYP3A4 inhibitors (e.g. ketoconazole, erythromycin, flucoconazole, voriconazole, clarithyomycin, amiodarone, telithryomycin)
- antibiotics such as erythryomycin, levofloxacin, moxifloxacin, spriamycin
- anti-fungal agents such as pentamidine
- anti-malarial agents such as halofantrine, lumefantrine
- gastro-intestinal medicines such as cisapride, dolasetron, prucalopride
- antihistamines (e.g. mequitazine, mizolastine)
- any cancer treatments (e.g. toremifene, vandetanib, nivamine)
- and AIDs/HIV medications (e.g. protease inhibitors)
- any antacids or antisecretory agents
