# Supplementary material for: Effects of Parkinson’s disease and dopamine on digit span measures of working memory
Source: Psychopharmacology (Berl). 2018 Oct 12;235(12):3443–50. doi: 10.1007/s00213-018-5058-6 (PMC6267128; doi:10.1007/s00213-018-5058-6)
Supplement: Supplementary file 2 — 2 Supplementary analyses including the percentage of digits correct, means and standard deviations of summary measures, and correlations between digit span measures and additional measures. (DOCX 968 kb) [file 213_2018_5058_MOESM2_ESM.docx]

Supplementary Materials 2:

Effects of Parkinson’s disease and dopamine on digit span measures of working memory

*Psychopharmacology*

John Patrick Grogan^1^, Lisa Emily Knight^2^, Laura Smith^1^, Nerea Irigoras Izagirre^1^, Alexandra Howat^1^, Brogan Elizabeth Knight^3^, Anastasia Bickerton^1^, Hanna Kristiina Isotalus^1^, Elizabeth Jane Coulthard^1,3^.

1. University of Bristol
2. University Hospitals Bristol
3. North Bristol NHS Trust

Correspondence to: John Grogan. Bristol Brain Centre, Elgar House, Southmead Hospital, Bristol, UK, BS10 5NB. +44 (0)1174148186. [John.grogan@bristol.ac.uk](mailto:John.grogan@bristol.ac.uk).

# Percentage of Digits Correct

We looked at the percentage of digits correct, which had many missing data, as the exact responses were not recorded for all participants. Only participants who had all the exact errors recorded could be included in the analysis of the percentage of digits correct, which meant only 45 PD OFF, 47 PD ON and 52 HC were included from Experiment 1. Two participants from Experiment 2 had all their errors recorded, but they were not analysed.


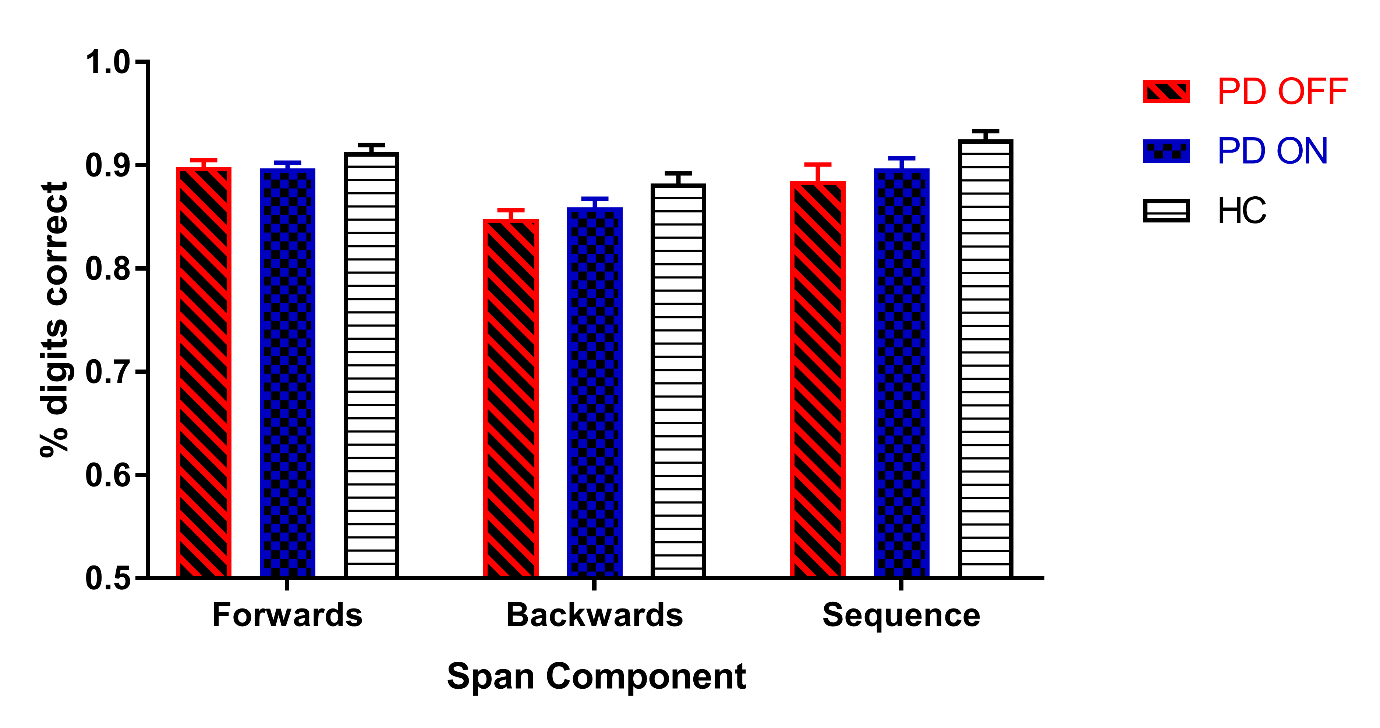


**Fig S1** Mean percentage of digits correct on each component of the digit span (SEM bars). Experiment 2 data were not analysed for this measure as only two participants had all their digit errors recorded. No comparisons passed the Bonferroni-corrected threshold.

PD patients did not have significantly lower percentage of digits correct than HC for forwards (F (2, 141) = 1.355, p = .261, $\eta_{p}^{2}$ = .019), backwards (F (2, 141) = 3.779, p = .025, $\eta_{p}^{2}$ = .051) or sequence (F (2, 141) = 3.498, p = .033, $\eta_{p}^{2}$ = .047) components. Backwards and sequence components did have larger effect sizes than the forwards span and the p-values were below .05 but did not pass the Bonferroni-corrected threshold (α = .01667). This suggests the pattern was the same as the percentage of lists correct, but with weaker effects due to the smaller sample size. Dopaminergic medication did not affect the percentage of digits correct on any of the digit span components (forwards: t (43) = -0.308, p = .760, *d* = 0.039; backwards: t (50) = 1.418, p = .162, *d* = 0.173; sequence: t (47) = 0.902, p = .372, *d* = 0.128).

**Table S1** Effect sizes and p-values from group comparisons of the percentage of digits correctly recalled. Between-subject one-way ANOVAs were used to compare HC vs PD ON vs PD OFF, while paired t-tests were used to compare PD ON vs PD OFF. Drug vs Placebo in Experiment 2 was not analysed as there were only 2 participants with error data. Bonferroni corrections were applied at a significance threshold of α=.01667. *p < .0167, **p < .00167, ***p < .000167.

| Comparison | Forwards | | Backwards | | Sequence | |
| --- | --- | --- | --- | --- | --- | --- |
|  | *d* | p value | *d* | p value | *d* | p value |
| HC vs PD-ON vs PD-OFF | 0.278 | 0.261282 | 0.464 | 0.025197 | 0.444 | 0.032902 |
| PD-ON vs PD-OFF | 0.039 | 0.759613 | 0.173 | 0.162338 | 0.128 | 0.371824 |
| Drug vs Placebo | N/A | N/A | N/A | N/A | N/A | N/A |

# Summary statistics of Results

Tables S2-S4 show the summary statistics for the measures of Maximum Span capacity, percentage of lists correct, and percentage of digits correct, respectively. These are the data plotted in Fig 1, Fig 2 and Fig S1, albeit using SEM rather than SD.

Table S2. Summary statistics for the Maximum Span Capacity measure for all groups (see Fig 1). Means, standard deviations (SD) and number of participants (N) are all shown.

| Comparison | Forwards | | Backwards | | Sequence | |
| --- | --- | --- | --- | --- | --- | --- |
|  | N | Mean (SD) | N | Mean (SD) | N | Mean (SD) |
| HC | 83 | 7.01 (1.19) | 83 | 5.08 (1.40) | 83 | 6.40 (1.32) |
| PD ON | 68 | 6.53 (1.40) | 68 | 4.37 (1.45) | 68 | 5.62 (1.56) |
| PD OFF | 68 | 6.38 (1.48) | 68 | 4.41 (1.26) | 68 | 5.54 (1.45) |
| Placebo | 30 | 7.07 (1.39) | 30 | 5.13 (1.28) | 30 | 6.40 (1.13) |
| Drug | 30 | 7.17 (1.49) | 30 | 5.20 (1.35) | 30 | 6.37 (1.22) |

Table S3. Summary statistics for the Percentage of Lists Correct measure for all groups (see Fig 2). Means, standard deviations (SD) and number of participants (N) are all shown.

| Comparison | Forwards | | Backwards | | Sequence | |
| --- | --- | --- | --- | --- | --- | --- |
|  | N | Mean (SD) | N | Mean (SD) | N | Mean (SD) |
| HC | 83 | 78.97 (6.13) | 83 | 75.52 (7.18) | 83 | 74.01 (7.82) |
| PD ON | 68 | 76.80 (7.85) | 68 | 72.59 (7.26) | 68 | 70.13 (10.50) |
| PD OFF | 68 | 77.89 (6.97) | 68 | 70.54 (7.32) | 68 | 68.24 (12.14) |
| Placebo | 30 | 79.25 (9.29) | 30 | 73.50 (7.10) | 30 | 75.26 (6.47) |
| Drug | 30 | 78.53 (10.46) | 30 | 72.84 (8.40) | 30 | 70.65 (6.91) |

Table S4. Summary statistics for the Percentage of Digits Correct measure for all groups (see Fig S1). Means, standard deviations (SD) and number of participants (N) are all shown.

| Comparison | Forwards | | Backwards | | Sequence | |
| --- | --- | --- | --- | --- | --- | --- |
|  | N | Mean (SD) | N | Mean (SD) | N | Mean (SD) |
| HC | 58 | 91.22 (4.43) | 58 | 88.63 (6.34) | 57 | 92.68 (5.27) |
| PD ON | 49 | 89.68 (4.21) | 52 | 85.90 (6.23) | 51 | 89.70 (6.97) |
| PD OFF | 49 | 89.85 (4.63) | 53 | 84.81 (6.36) | 52 | 88.47 (11.60) |
| Placebo | 3 | 95.75 (4.02) | 2 | 84.75 (7.42) | 2 | 91.31 (5.56) |
| Drug | 6 | 88.10 (6.15) | 5 | 82.52 (6.41) | 4 | 92.00 (4.06) |

# Dose-Dependent Associations

## Experiment 1

We found no linear or polynomial correlations between the change in digit span maximum capacity (each component) or percentage of lists correct and levodopa dose equivalency (LDE). For each component, we fit a linear and quadratic line using least-squares difference, and then compared these two fits using an F test. The best fitting of these was then accepted if the p-value was below the Bonferroni-corrected threshold, which did not happen for any of the associations tested (p > .01667).

There was a trend linear association between LDE and percentage of digits recalled correctly backwards (p = .0175) although this did not pass the Bonferroni-corrected threshold.


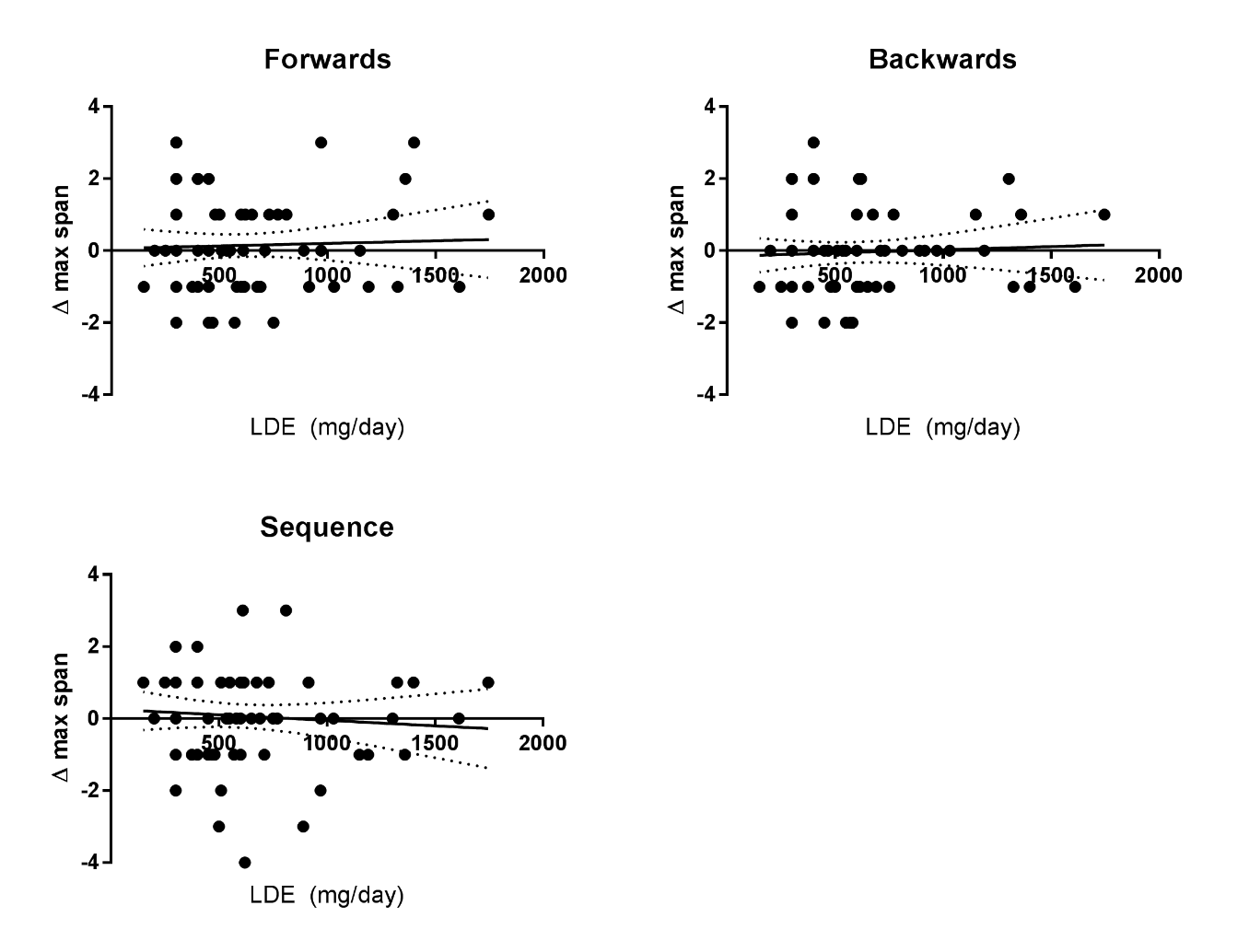


**Fig S2** Levodopa Dose Equivalency (LDE) was not significantly associated wih the difference in maximum digit span between PD ON and OFF for any component (linear or quadratic). Here are shown the best fitting lines which were linear in all occasions (dotted lines show 95% Confidence Intervals), although none were significant fits (p > .01667).


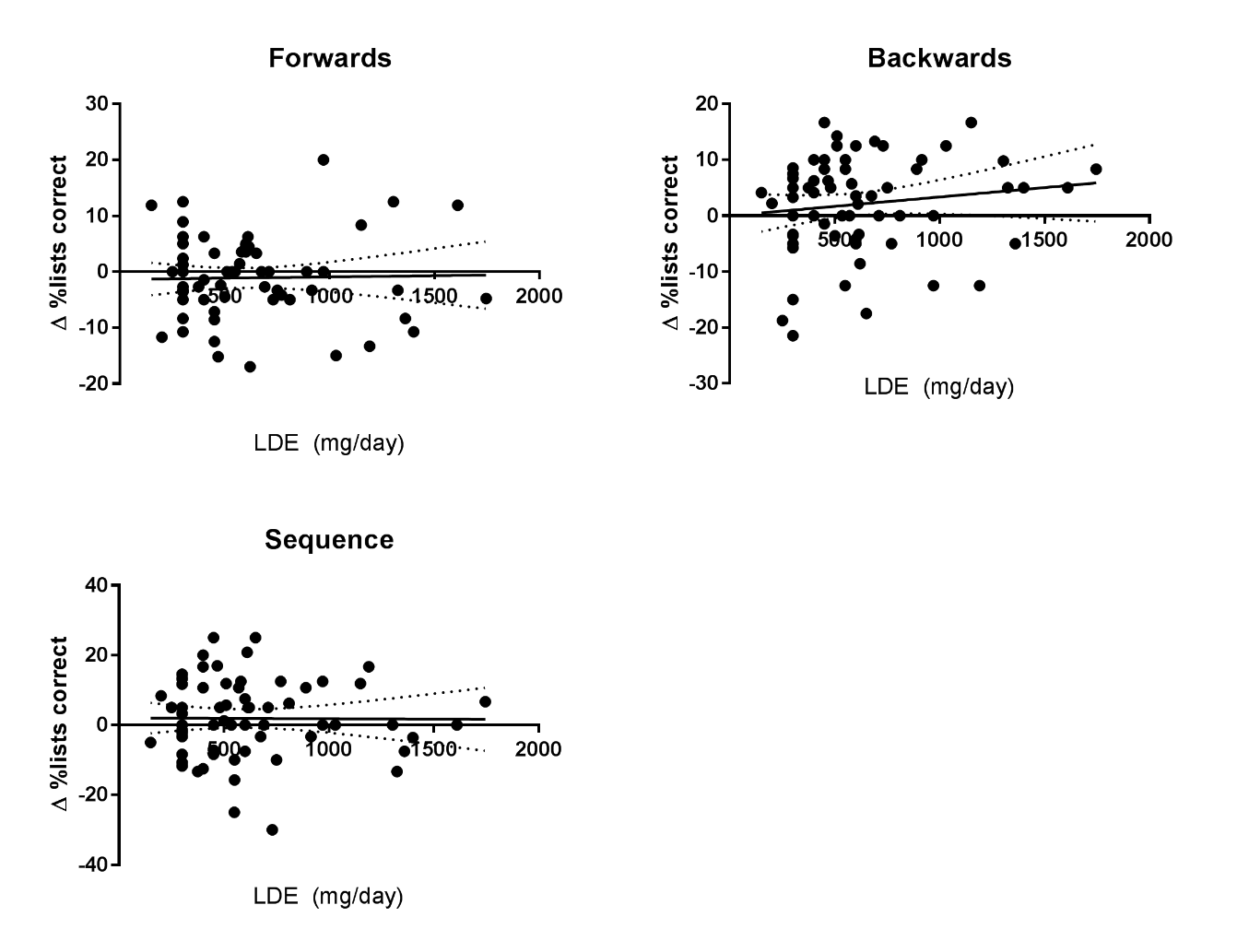


**Fig S3** The difference in percentage of lists correct between PD ON and PD OFF was not correlated with LDE for any digit span component (linear or quadratic). Here are shown the best fitting lines which were linear in all occasions (dotted lines show 95% Confidence Intervals), although none were significant fits (p > .01667).


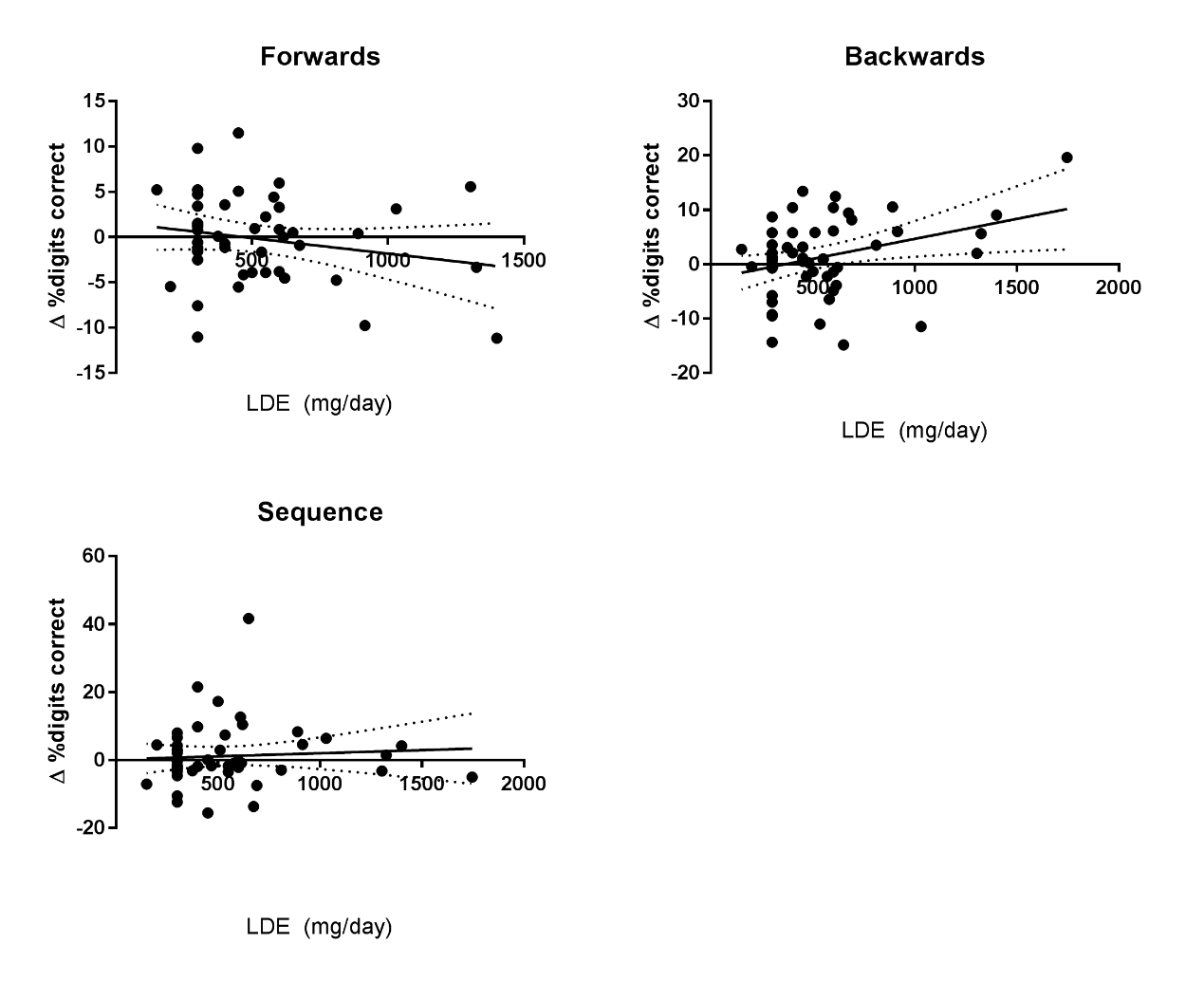


**Fig S4** The difference in percentage of lists correct between PD ON and OFF was not correlated with LDE for any digit span component (linear or quadratic). Here are shown the best fitting lines which were linear in all occasions (dotted lines show 95% Confidence Intervals), although none were significant fits (p > .01667).

We also found no interactions when running ANCOVAs with LDE as a covariate on the maximum spans (p > .5), percentage of lists correct (p > .2) or digits correct (p > .25).

We also included medication type (levodopa only, agonists only or both) and side of symptom onset as between-subject factors (separately) which also had no effect on maximum spans (p > .2, p > .09), percentage of lists correct (p > .2, p > .25) or digits correct (p > .4, p > .03).

## Experiment 2

We divided the levodopa dose (150mg) by the participants’ weight (kg) to give relative levodopa dose (mg/kg) and looked for associations between this and performance on the digit span, to see if people who had larger doses scored worse on the span due to more ‘overdosing’ of the intact WM processes. We found no linear or polynomial associations (p > .01667).


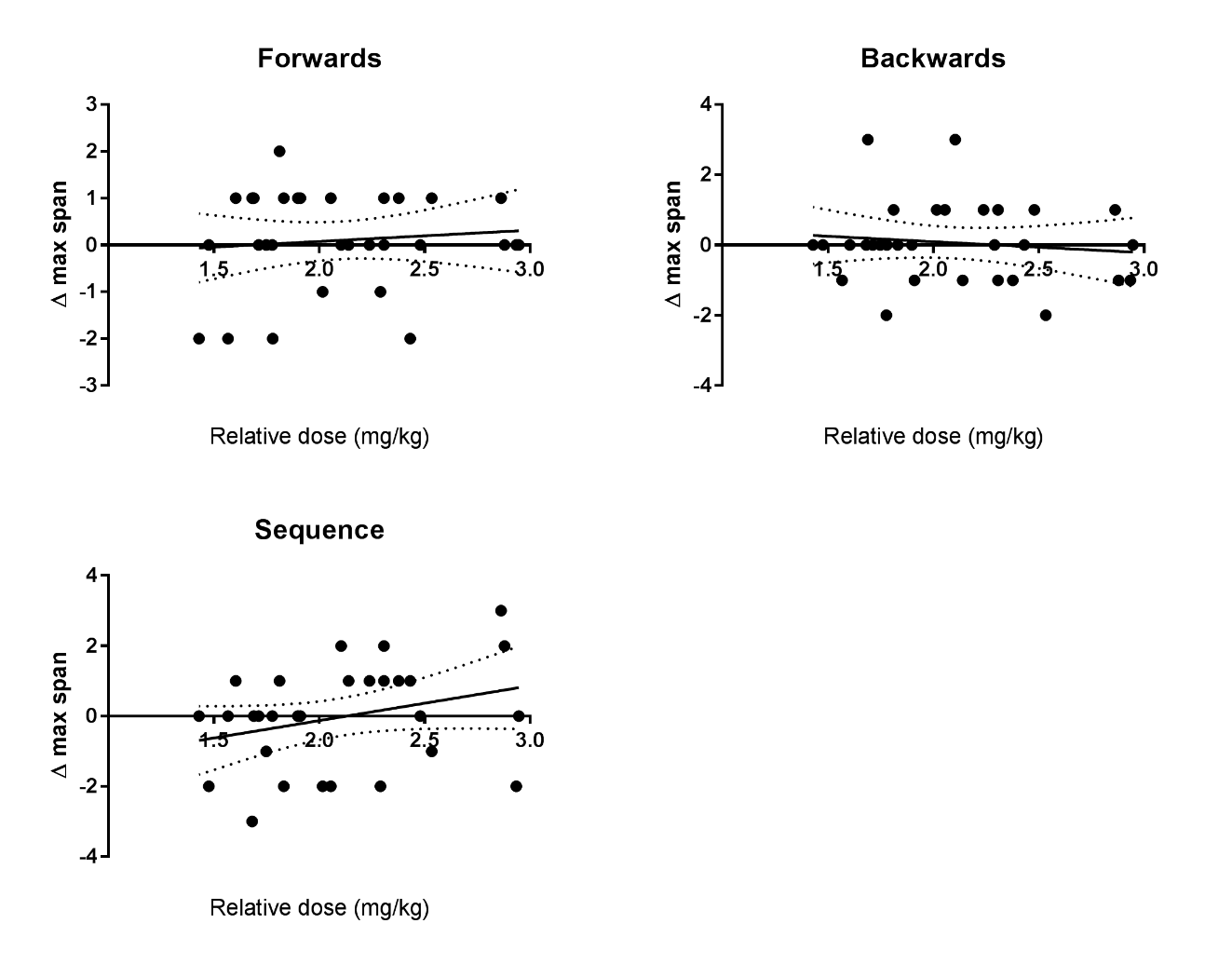


**Fig S5** Relative levodopa dose was not associated with the difference in maximum digit span between drug and placebo conditions for any component (linear or quadratic). Here are shown the best fitting lines which were linear in all occasions (dotted lines show 95% Confidence Intervals), although none were significant fits (p > .01667).


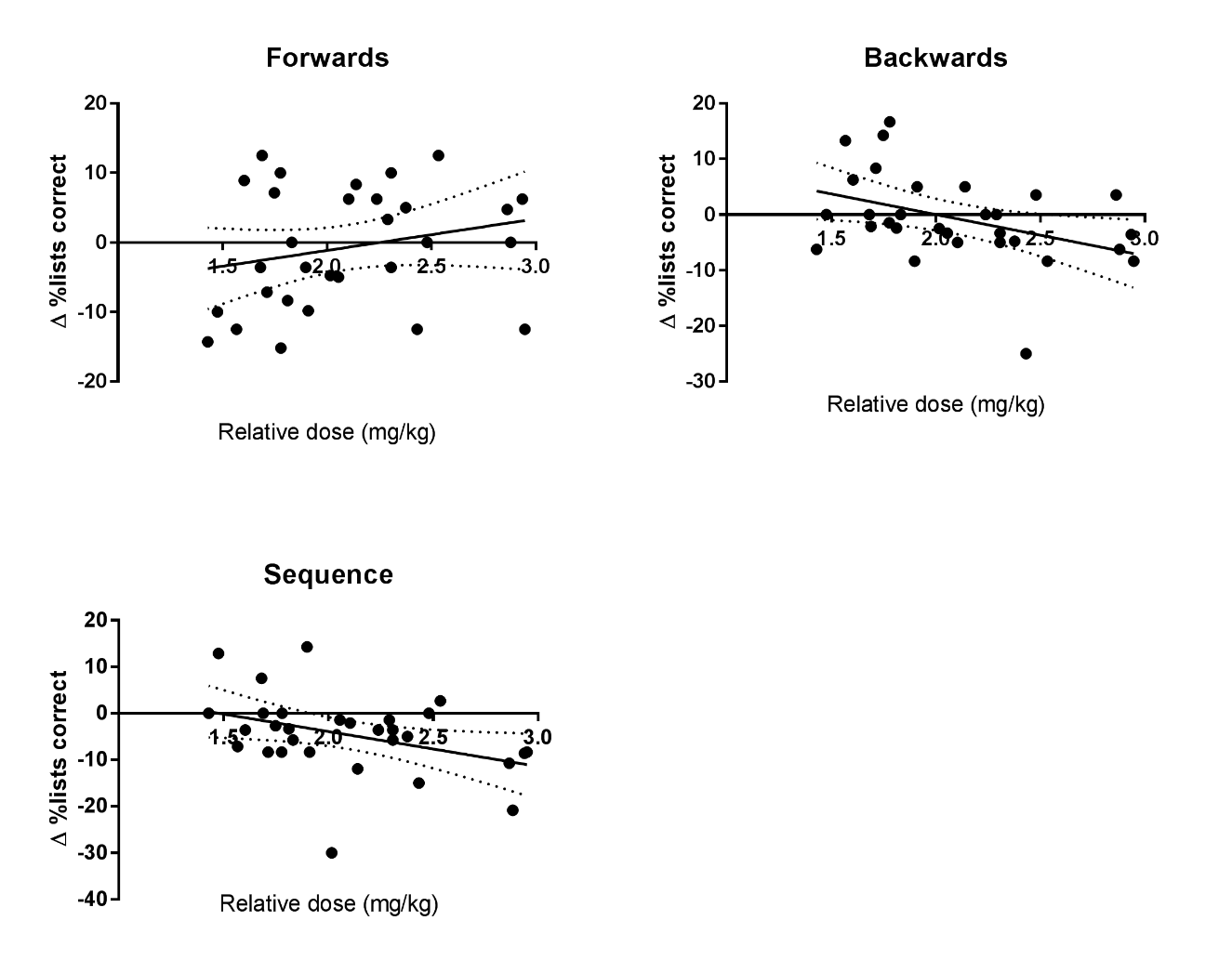


**Fig S6** Relative levodopa dose was not associated with the difference in percentage of lists correct between drug and placebo conditions for any component (linear or quadratic). Here are shown the best fitting lines which were linear in all occasions (dotted lines show 95% Confidence Intervals), although none were significant fits (p > .01667).

We also included relative dose as a covariate in an ANCOVA which did not interact with drug condition or change the results for maximum spans (p > .1) or percentage of lists correct (p > .02).

# Correlations between Measures

The three measures we have been analysing are all generated from the same data, so we looked at the extent to which they are all correlated (see Table S5). There were weak to medium correlations between most of the measures, the strongest being between % list accuracy and % digits correct for the same component (e.g. backwards and backwards). Some of the correlations with % digits correct for forwards and sequence components were not significant (p > .01667).

Table S5 The Pearson’s correlation coefficients between the different components (F=Forwards, B=Backwards, S=Sequence) for the three digit span measures, across all participant groups. *p < .0167, **p < .00167, ***p < .000167.

|  | | Capacity | | | % Lists correct | | | % Digits Correct | | |
| --- | --- | --- | --- | --- | --- | --- | --- | --- | --- | --- |
|  |  | F | B | S | F | B | S | F | B | S |
| Capacity | F |  |  |  |  |  |  |  |  |  |
|  | B | 0.5013*** |  |  |  |  |  |  |  |  |
|  | S | 0.3942*** | 0.4510*** |  |  |  |  |  |  |  |
| % Lists Correct | F | 0.5898*** | 0.3885*** | 0.3184*** |  |  |  |  |  |  |
|  | B | 0.3173*** | 0.4384*** | 0.3625*** | 0.3221*** |  |  |  |  |  |
|  | S | 0.3209*** | 0.3687*** | 0.5068*** | 0.1921** | 0.2626*** |  |  |  |  |
| % Digits Correct | F | 0.2346* | 0.3141*** | 0.1683 | 0.6950*** | 0.2852*** | 0.1796 |  |  |  |
|  | B | 0.3372*** | 0.4907*** | 0.3633*** | 0.2963*** | 0.8029*** | 0.2339* | 0.2626*** |  |  |
|  | S | 0.3221*** | 0.3326*** | 0.4365*** | 0.1268 | 0.1693 | 0.7499*** | 0.0892 | 0.2617*** |  |

# Individual Differences

Post-hoc analyses were conducted to examine the influence several differences between PD patients may have had on their WM performance. These included duration of disease and cognitive performance (MoCA score).

Years since diagnosis was used for a median split (median = 3 years), and this early/late grouping was entered as a between subject factor into repeated measures ANOVAs comparing performance ON and OFF medication for PD patients, to see whether early and late PD had different responses to medication (i.e. early/late * medication interaction). Please note that the main text used t-tests to compare ON and OFF medication which do not allow for inclusion of between subject factors, although a repeated measures ANOVA and t-tests give the same results when medication state is the only within-subject factor. There were no significant interactions of early/late PD and medication state for any component on any of the three measures used (p > .1).

Similarly, MoCA was considered. We split all participants into high or low cognitive function using a MoCA cut-off of < 26 for low function (Hoops et al., 2009; Nasreddine et al., 2005). As this was present for PD and HC, we performed two sets of analyses, one to look for a MoCA * group interaction in a between subject ANOVA, and one to look for a MoCA * medication interaction in a repeated measures ANOVA just in PD patients. We found no interactions between cognitive function and group for any component or measure (p > .1), nor and cognitive function * medication state interactions in PD patients (p > .01667).

We also looked for correlations between the other questionnaire data we collected and the difference between patients’ scores ON and OFF medication. We found no significant associations between accuracy or capacity for any component with the DASS, BIS, LARS, MoCA, disease duration, or UPDRS score ON or OFF (p > .0167).
