# Supplementary material for: Effects of Parkinson’s disease and dopamine on digit span measures of working memory
Source: Psychopharmacology (Berl). 2018 Oct 12;235(12):3443–50. doi: 10.1007/s00213-018-5058-6 (PMC6267128; doi:10.1007/s00213-018-5058-6)
Supplement: Supplementary file 3 — 3 Procedure for scoring error types on the digit span, and analysis of these error types. (DOCX 454 kb) [file 213_2018_5058_MOESM3_ESM.docx]

Supplementary Materials 3:

Effects of Parkinson’s disease and dopamine on digit span measures of working memory

*Psychopharmacology*

John Patrick Grogan^1^, Lisa Emily Knight^2^, Laura Smith^1^, Nerea Irigoras Izagirre^1^, Alexandra Howat^1^, Brogan Elizabeth Knight^3^, Anastasia Bickerton^1^, Hanna Kristiina Isotalus^1^, Elizabeth Jane Coulthard^1,3^.

1. University of Bristol
2. University Hospitals Bristol
3. North Bristol NHS Trust

Correspondence to: John Grogan. Bristol Brain Centre, Elgar House, Southmead Hospital, Bristol, UK, BS10 5NB. +44 (0)1174148186. [John.grogan@bristol.ac.uk](mailto:John.grogan@bristol.ac.uk).

# Error types analysis

We examined the different types of errors that participants made on the digit span.

The exact responses were only recorded for some participants (45 PD and 52 HC from Experiment 1, and only for two participants from Experiment 2), which were the only participants we could apply this analysis to. Experiment 2 data were not analysed as only 2 participants had errors recorded.

We adapted an automated marking scheme (Woods et al., 2011) for manual marking, and the exact steps used are shown at the end of this document. In brief, we first counted how many digits were correct-in-position, and then looked for transposition (order) errors, and then number errors (e.g. substitution, omission, intrusion) among the remaining digits.

There were 3 types of transposition errors: 1^st^ order, where consecutive digits are swapped (e.g. 1234 becomes 1324); 2^nd^ order, where digits separated by 1 digit are swapped (1234 becomes 1432); permutation errors, which are any order errors not easily explained by the former two (1234 becomes 3142). Number errors could be: substitutions, where a digit is changed for another (1234 -> 1239); omissions, where a digit is dropped (1234 -> 124); or intrusions, where a digit is added (1234 -> 12384).

As participants frequently gave incomplete responses, we used several ‘partial attempt’ errors to prevent these over-inflating the number of omission errors. If a person recalled < 50% of the list or made 3 consecutive omissions in the middle of the list it was ‘partial attempt 1’, if they omitted 2 or more consecutive digits at the beginning or end of the list it was a partial attempt 2 or 3, respectively. There was also a ‘did not attempt’ if no digits were said, and an ‘unclassifiable response’ if the response was so dissimilar to the list that it was unable to be explained simply using the errors above.

If there were multiple possible interpretations of the errors, the one that resulted in fewest errors was used. If there were still multiple possibilities, priority was given to explanations using order errors over number errors.

We analysed the number of order errors in total by summing the number of transposition errors and permutation errors. We also looked at the number of omissions, intrusions, and substitutions, separately, for each digit span component. The sequence span should be considered separately as order errors mean something different here – they are not the result of misbinding the digit and its location, but rather an error in the order in which digits should be recalled. There were very few order errors in the sequence component, but they may reflect a different process to order errors in the other two components.

# Results

We ran one-way ANOVAs on the four error types, with condition (ON, OFF, HC) as a between subject factor, and a Bonferroni-corrected threshold of α = .0167. Order errors (Fig S7) were significantly higher in PD patients than HC only for the backwards component (F (2, 161) = 8.177, p = .0004, $\eta_{p}^{2}$ = .092), but not for the forwards (F (2, 161) = 1.064, p = .347, $\eta_{p}^{2}$ = .013) or backwards components (F (2, 161) = 2.159, p = .119, $\eta_{p}^{2}$ = .026). Bonferroni-corrected post-hoc comparisons showed this was due to both patients ON and OFF having more order errors than controls (p = .001 & p = .004, respectively). Paired sample t-tests showed no difference between PD ON and OFF (forwards: t(51) = 1.670, p = .101; backwards: t(51) = .325, p = .746; sequence: t(51) = -.1.031, p = .307).


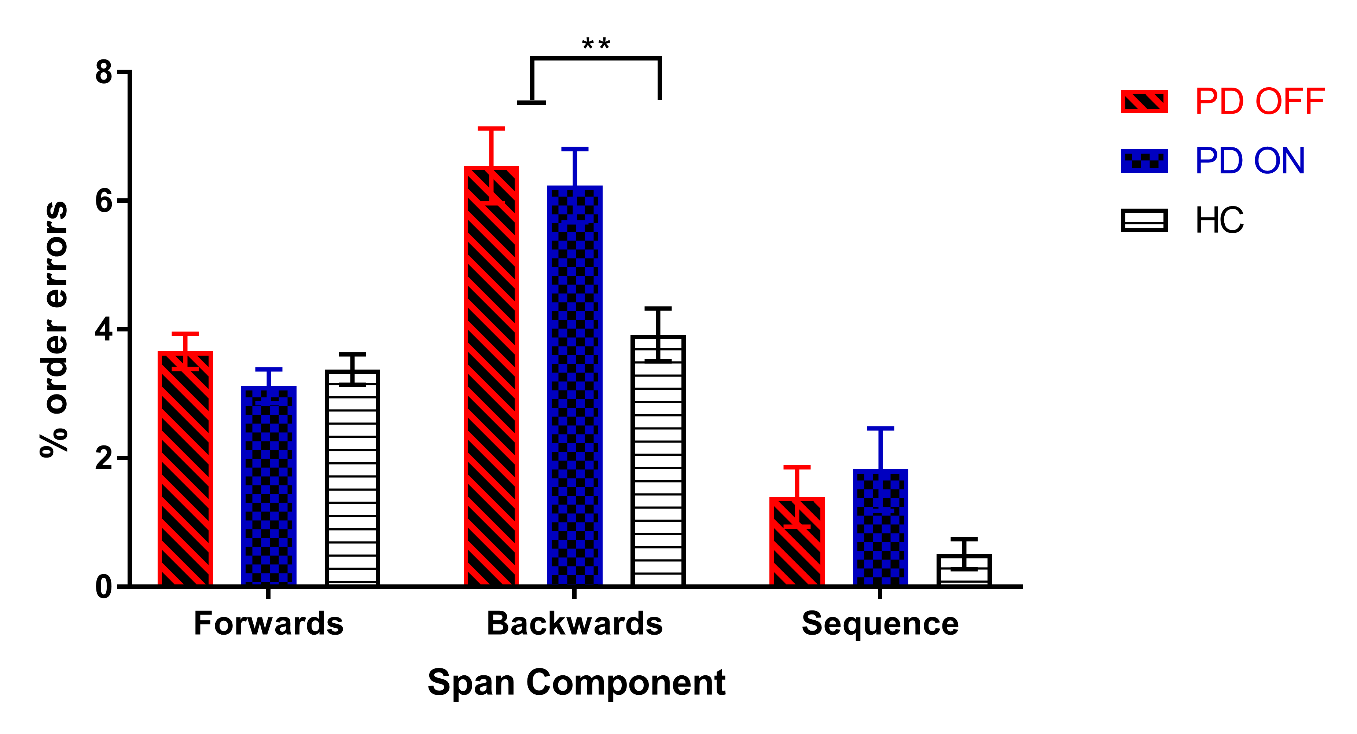


**Figure S7** The percentage of digits with order errors (1^st^ or 2^nd^ order transposition errors or permutation errors) for each digit span component. ** = p < .00167.

We also looked at each different order error separately, which showed that only the 1^st^ order transposition errors in the backwards component differed significantly (F (2, 161) = 4.453, p = .013, $\eta_{p}^{2}$ = .052), although the 2^nd^ order transposition errors had a similar pattern albeit with the backwards component effect not passing the Bonferroni-corrected significance threshold (p = .040; see Fig S8 and Table S6 for details). This may have been due to there being far fewer 2^nd^ order errors than 1^st^ order errors, which reduced sensitivity. Permutation errors had no differences between groups.


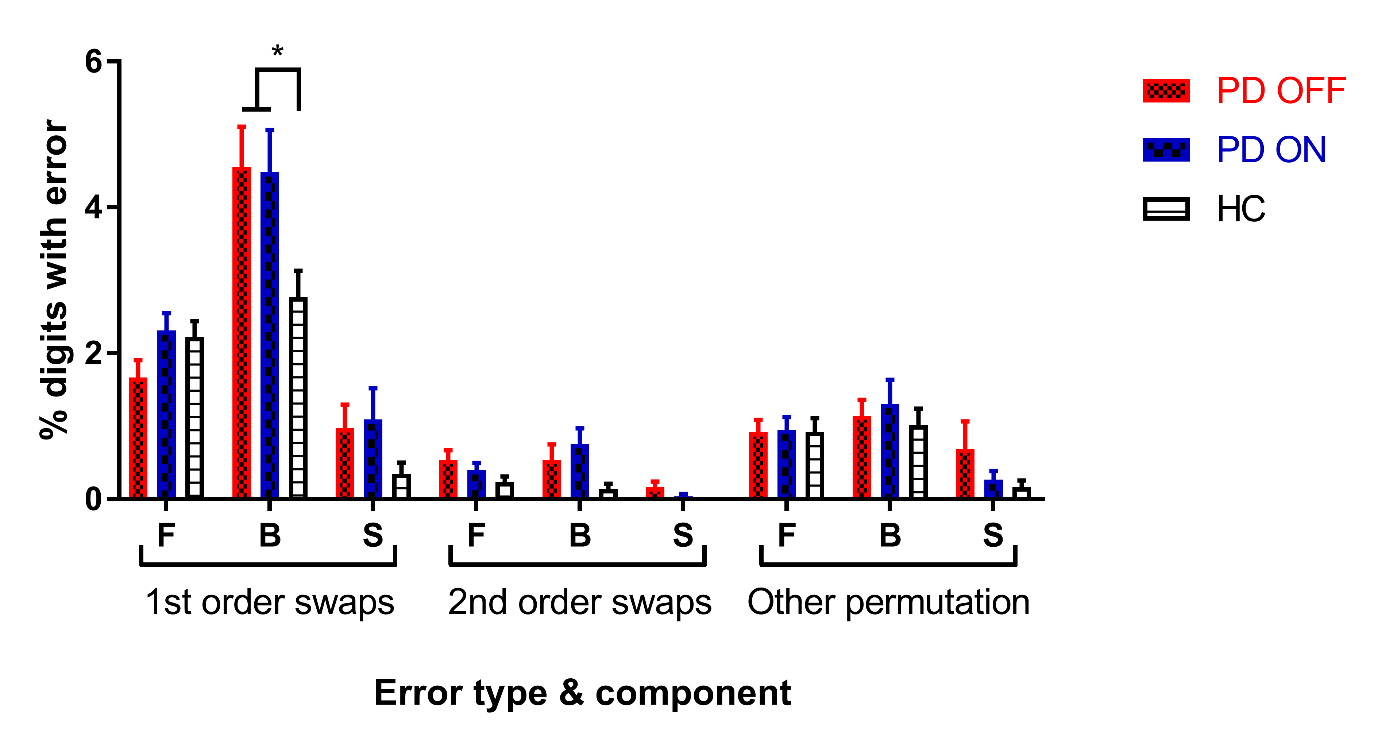


**Figure S8** The percentage of digits with each separate order error for each digit span component. * = p < .0167.

Omission, intrusion and substitution errors were no different between groups, or between medication conditions (p > .0167; see Fig S9 and Table S6 for statistics).


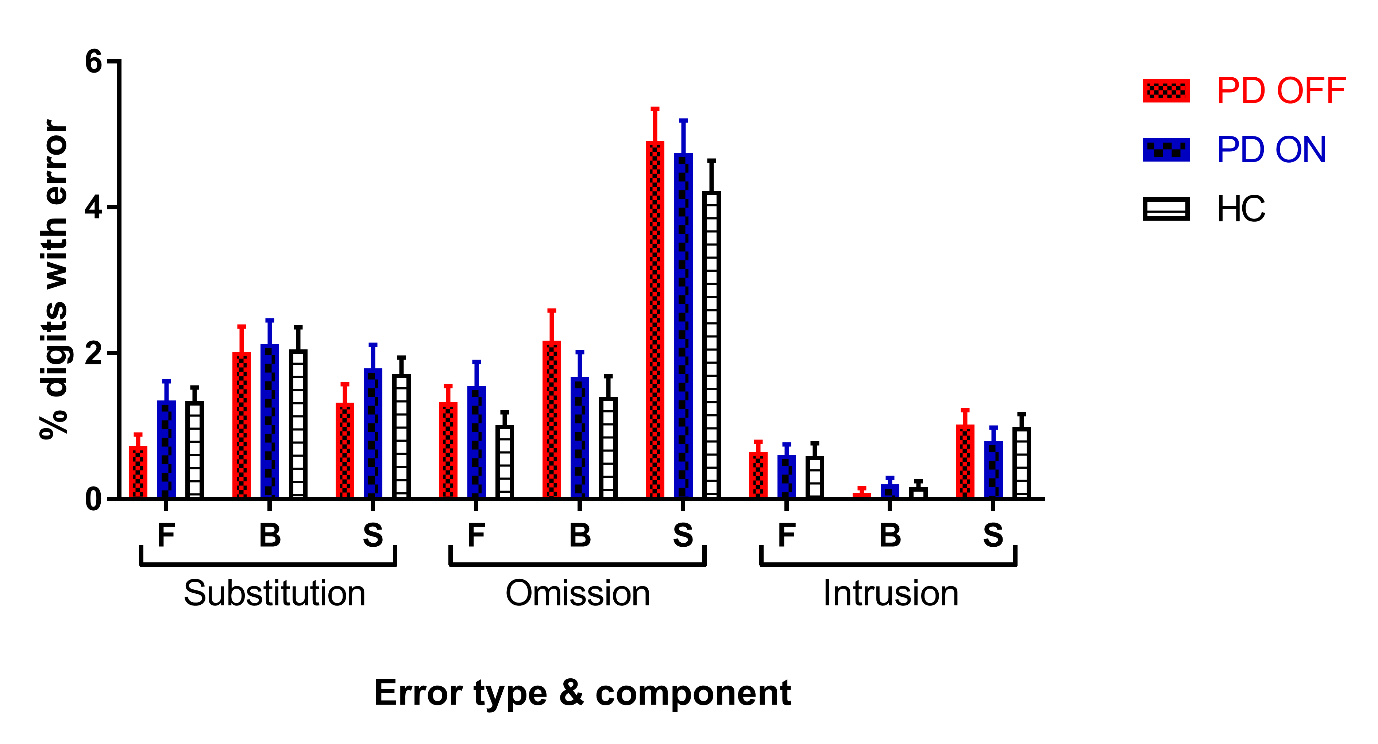


**Figure S9** The percentage of digits with substitution, omission, or intrusion errors for each digit span component. There were no significant differences between the groups.

Table S6 P-values from between subject ANOVAs (Group comparison) and paired t-tests (PD Meds comparison) on the different error types. Bonferroni-corrected significance threshold: α = .0167. * = p < .0167, *** = p < .0001667.

| Error | Comparison | Forwards | Backwards | Sequence |
| --- | --- | --- | --- | --- |
| Order - all | Group | .347 | .0004*** | .119 |
|  | PD Meds | .101 | .746 | .307 |
| 1^st^ Order Swap | Group | .111 | .013* | .176 |
|  | PD Meds | .053 | .810 | .695 |
| 2^nd^ Order Swap | Group | .119 | .040 | .057 |
|  | PD Meds | .316 | .493 | .056 |
| Permutation | Group | .991 | .772 | .254 |
|  | PD Meds | .793 | .735 | .307 |
| Substitution | Group | .057 | .950 | .386 |
|  | PD Meds | .039 | .865 | .150 |
| Omission | Group | .309 | .324 | .559 |
|  | PD Meds | .495 | .423 | .754 |
| Intrusion | Group | .967 | .797 | .764 |
|  | PD Meds | .734 | .341 | .396 |

This suggests that PD patients make more order errors only on the backwards component, and otherwise have no clear differences in error types. As mentioned earlier, order errors are different in the sequence span as they do not result from mistaking the order the item was presented in, but rather a mistake in the order they were meant to recall them in (i.e. as order errors in sequence span must be, by definition, in descending order rather than ascending). Therefore, it is not surprising that there was an effect of PD on one manipulation component (backwards) but not the other (sequence). This suggests that increasing the manipulation required in WM leads to more order errors for PD patients than HC, but this deficit is hidden if participants are required to recall the items in an order unrelated to their presentation order (i.e. ascending order).

# Error marking procedure

*Modified from Woods, Herron, Yund, Hink, Kishiyama and Reed (2011) “Computerised analysis of error patterns in digit span recall” (error types OE, IE, SE, TE1, TE2, PE are the same error types, error types PA1, PA2, PA3 and DNA have been added).*

## Error Types

First order Transposition Errors (TE1): The order of two digits immediately next to each other are transposed e.g. if the string is 1-2-3-4 the participant reports 1-3-2-4

Second order Transposition Errors (TE2): The order of two digits separated by another digit are transposed e.g. if the string is 1-2-3-4 the participant may report 1-4-3-2

Substitution Errors (SE): A digit is incorrectly substituted with another digit e.g. if the string is 1-2-3-4- the participant reports 1-2-7-4

Omission Errors (OE): A digit is forgotten e.g. if the string is 1-2-3-4 the participant reports 1-2-X-4. A “Don’t know” or “something” response counts as an omission.

Intrusion Errors (IE): A digit is added in e.g. if the string is 1-2-3-4 the participant reports 1-2-7-3-4

Permutation Errors (PE): Any order error of three or more digits that can’t be explained easily by TE1 or TE2. This includes digits in the wrong order, or transpositions with more than 1 digit in-between the swapped digits.

Partial attempt (PA) (for PA2&3: mark whatever is said with whatever errors are there, and use PA for the missing parts):

1. Participant recalls less than 50% of the string or makes 3 or more consecutive omission errors in the middle of the string e.g. if the string is 1-2-3-4 the participant reports X-2-X-X or e.g.
2. Participant omits two or more consecutive digits at the beginning of the string e.g. if the string is 1-2-3-4 the participant reports X-X-3-4
3. Participant omits two or more consecutive digits at the end of the string e.g. if the string is 1-2-3-4 the participant reports 1-2-X-X

Did not attempt (DNA): Participant does not attempt the string.

Unclassifiable Response (UR): Too complex to explain in terms of error types above.

## Protocols for classifying errors

- Self-corrections will not be classified as errors.
- When a string has been recalled incorrectly the error types should be classified in such a way that minimises the number of errors made. e.g. if a string 1-2-3-4-5-6 was recalled as 4-3-2-5-6 this could be classified as:

1. A substitution, a first order transposition error and an omission: 4(substitution for 1)-3(first order transposition error)-2 (first order transposition error)-X (omission of 4)- 5-6; which would give 3 errors (this is one example of many!)
2. OR An omission and a second order transposition: X (omission of 1)-4(2 second order transposition error)-3-2(4 second order transposition error)-5-6 which would give two errors.

In this case, the 2^nd^ option would be chosen as it has fewer errors.

- If a string is recalled in such a way that it may be interpreted in a number of different ways, which all give the same number of errors, errors should be prioritised in the order that they’re listed above.
- For the backwards and sequence digit spans errors are considered in comparison to the correct response for example if the sequence 1-2-3-4-5 in the backwards condition was recalled as 5-4-3-1-2 this would be a first order transposition error as the correct response should be 5-4-3-2-1. Similarly if 3-2-4-1-5 in the sequence span was recalled as 2-3-4-5 this would be classified as on omission error as the correct response should be 1-2-3-4-5.
